# Porcine induced pluripotent stem cell-derived osteoblast-like cells prevent glucocorticoid-induced bone loss in Lanyu pigs

**DOI:** 10.1371/journal.pone.0202155

**Published:** 2018-08-29

**Authors:** Yu-Jing Liao, Pin-Chi Tang, Yu-Hsin Chen, Feng-Hsiang Chu, Ting-Chieh Kang, Lih-Ren Chen, Jenn-Rong Yang

**Affiliations:** 1 Division of Physiology, Livestock Research Institute, Council of Agriculture, Executive Yuan, Tainan, Taiwan; 2 Department of Animal Science, National Chung Hsing University, Taichung, Taiwan; 3 The iEGG and Animal Biotechnology Center, National Chung Hsing University, Taichung, Taiwan; 4 Center for the Integrative and Evolutionary Galliformes Genomics, National Chung Hsing University, Taichung, Taiwan; 5 Department of Animal Science, National Chiayi University, Chiayi, Taiwan; 6 Hengchun Branch, Livestock Research Institute, Council of Agriculture, Executive Yuan, Tainan, Taiwan; 7 Graduate Institute of Bioresources, National Pingtung University of Science and Technology, Pingtung, Taiwan; 8 Department of Biotechnology and Bioindustry Sciences, National Cheng Kung University, Tainan, Taiwan; Van Andel Institute, UNITED STATES

## Abstract

The application of appropriate animal models and techniques for the study of osteoporosis is important. Lanyu pigs, a local miniature breed, have been widely used in various biomedical studies in Taiwan. This study aimed to induce bone loss in Lanyu pigs and to examine whether porcine induced pluripotent stem cell (piPSC)-derived osteoblast-like cells could recover bone mass of tibiae via local cell transplantation. piPSCs were directed to differentiate into osteoblast-like cells using osteogenic medium, and differentiated cells expressed osteogenic markers and phenotypes. Twenty mature female Lanyu pigs were divided into four groups, including control (C, 1% calcium diet), treatment 1 (T1, ovariectomy + 1% calcium diet), treatment 2 (T2, ovariectomy + 0.5% calcium diet), and treatment 3 (T3, ovariectomy + 0.5% calcium diet + 1 mg/kg of prednisolone) and were subjected to bone loss induction for twelve months. Micro-CT images revealed that the lowest trabecular bone parameters, such as trabecular bone volume, thickness, separation, number, and total porosity, were detected in the T3 group. The lowest proportions of cortical bone in the proximal metaphysis, proximal diaphysis, and distal diaphysis were also found in the T3 group. These results indicate that ovariectomy, calcium restriction, and prednisolone administration can be applied to induce proper bone loss in Lanyu pigs. After bone loss induction, pigs were subjected to cell transplantation in the left tibiae and were maintained for another six months. Results showed that transplanted piPSC-derived osteoblast-like cells significantly improved trabecular bone structures at transplanted sites and maintained cortical bone structures in the proximal metaphysis. In conclusion, the therapeutic potential of piPSC-derived osteoblast-like cells was confirmed via cell transplantation in the left tibiae of Lanyu pigs. These findings reveal the therapeutic potential of piPSCs for glucocorticoid-induced bone loss in pig models.

## Introduction

Osteoporosis is a prevalent health problem characterized by a series of serious bone disorders. The application of appropriate animal models and techniques for osteoporosis study is important in understanding its pathogenesis, progression, and therapy [1]. Rodents have the characteristics of anatomical structures, bone remodeling, bone loss caused by estrogen deficiency in common with humans and have been animal models in osteoporosis research for several decades. However, the genetic and physiological differences to humans caused by body size should be concerned when compared with other larger mammalian species [1]. Comparatively, pigs also have several characteristics in common with humans, such as organ size, anatomical structures, bone remodeling, bone loss caused by estrogen deficiency, and non-seasonal estrous cycle [2–6]. Due to the body size of mini pigs, they are suitable for use in long-term studies and for replacement of rodents in osteoporosis research [1]. The Lanyu pig used in the present study is an indigenous miniature breed originating from Lanyu Islet southeast of Taiwan, and they have distinctive phenotypic characteristics compared with other pig breeds in Asia and Europe [7]. After introduction and selective inbreeding at Taitung Animal Propagation Station of Livestock Research Institute since 1980, the Association for Assessment and Accreditation of Laboratory Animal Care (AAALAC) International accredited Lanyu pigs have been widely used in biomedical research in Taiwan.

Several experimental interventions have been used to induce osteoporosis in rats, such as hormonal interventions, dietary interventions, and immobilization [8]. Among these experimental interventions, either ovariectomy or glucocorticoid treatment is a typical method [9]. Calcium is required for regulation of bone remodeling and bone mass [10], and long-term calcium deficiency also leads to osteoporosis [11]. Based on these theories, a method combining ovariectomy and a calcium-restricted diet has been used to develop osteoporosis in mini pigs [12]. Glucocorticoids are applied to inhibit autoimmune diseases and to prevent rejections during organ transplantation. However, long-term application of glucocorticoids also results in bone loss and low bone turnover [13–15]. These results have been confirmed in mini pigs that received prednisolone for more than one year; therefore, glucocorticoid treatment is an alternative strategy to induce osteoporosis in animal models [16].

Osteoclasts function in removal of mature bone tissue, and osteoblasts engage in formation of bone matrix. Both of these cell types maintain bone remodeling [17]. If their activity is imbalanced, bone loss and osteoporosis ensue [18]. The use of hormone replacement to induce bone formation or osteoclast suppression has been a method of osteoporosis treatment for decades [18]. Recently, stem cell therapy use in animal models of osteoporosis has been confirmed via local or systemic cell transplantation [9], and this therapy reduces susceptibility to fractures [19].

In the present study, we aimed to promote progression of tibial bone loss in Lanyu pigs and to induce osteoblast differentiation of porcine induced pluripotent stem cells (piPSCs). Thereafter, resultant deficiencies of microarchitecture in tibiae were determined and evaluated by micro-computed tomography (micro-CT) detection, three-point bending mechanical test, and mineral content analysis. Finally, we determined whether local transplantation of piPSC-derived osteoblast-like cells could prevent tibial bone loss in Lanyu pigs.

## Materials and methods

### *In vitro* culture of piPSCs

The method for piPSC induction was described as our previous study [20]. Briefly, porcine ear fibroblasts used in present study were collected from ear tissues of Livestock Research Institute Black Pig No. one. The ear tissues were trypsinized by 0.25% (w/v) trypsin-0.02 mM EDTA (Invitrogen, Grand Island, NY, USA) and were maintained in cell culture medium as described previously [20]. piPSCs expressing green fluorescent protein (GFP) were generated from porcine ear fibroblasts transfected with human *OCT4*, *SOX2*, *KLF4*, and *c-MYC* genes cloned into lentivirus vectors (TLC-TRE-iPS-II, Tseng Hsiang Life Science LTD, Taipei, Taiwan) and were maintained in embryonic stem cell (ESC) culture medium as described previously [20].

### *In vitro* induction of osteogenic differentiation of piPSCs

To induce differentiation of osteoblast-like cells, undifferentiated piPSCs were trypsinized into single cells and were seeded into fibronectin-coated 4-well plates. Cells were cultured in osteogenic medium containing MEM-α (Invitrogen, Grand Island, NY, USA), 10% fetal bovine serum (FBS) (Invitrogen), 50 μM L-ascorbic acid (Sigma-Aldrich, St. Louis, MO, USA), 10 mM β-glycerophosphate (Sigma-Aldrich), 100 nM dexamethasone (Sigma-Aldrich), 10 nM 1α,25(OH)2D3 (Sigma-Aldrich), and 40 ng/mL bone morphogenetic protein 2 (BMP2) (Invitrogen) for 4 weeks [21–23].

### Characterization of osteogenic phenotypes

To identify mineralization, cells were fixed in 10% neutral buffered formalin and stained with Alizarin Red S (Sigma-Aldrich). For Alizarin Red S staining, fixed cells were incubated with 2% Alizarin Red S solution (pH 4.2) for 5 min and later washed twice in deionized and distilled water [21,23].

### Characterization of osteogenic markers

For immunocytochemical staining, cells were fixed in 10% (v/v) neutral buffered formalin and stained with specific antibodies as described previously [20]. Primary antibodies used for staining included osteonectin (Cat. #SC-25574), osteocalcin (Cat. #SC-30044), and collagen type I (Cat. #SC-28657) (Santa Cruz Biotechnology, Inc., Dallas, TX, USA). Secondary antibodies were goat anti-rabbit IgG (Cat. #111-025-003; Jackson ImmunoResearch, West Baltimore Pike, PA, USA). All primary and secondary antibodies were diluted at a ratio of 1:100.

### Experimental animals

All animal experiments in the present study were performed in accordance with ethical guidelines following approval of the Livestock Research Institute Institutional Animal Care and Use Committee (IACUC, No. 105–31). Twenty female Lanyu pigs (Lanyu 200, http://minipigs.angrin.tlri.gov.tw/english/modules/tinyd2/) at 6 months of age and with a body weight between 24–37 kg were obtained from Taiwan Taitung Animal Propagation Station. Pigs were maintained at room temperature and were allowed free access to water and food for 1 week before treatments. At the end of experiments, pigs were sacrificed in the slaughterhouse (Tainan Meat Market Corporation, Annan Dist, Tainan, Taiwan) with standard slaughtering procedures.

### Ovariectomy

Prior to surgery, fifteen pigs were fasted for 12 hr. The animals were intramuscularly anesthetized with 8 mg/kg body weight of Zoletil^®^ 50 (Virbac corporation, Carros, France). The hair on the abdomen was completely removed by surgical scalpel blade. The skin of the abdomen was disinfected by 75% ethanol and iodine disinfectant before surgery. Ovariectomy was made by two ventral incisions. After identifying the ovary and uterine horn, a silk suture was performed around the area of the distal uterine horns, and the ovaries were removed. The wounds in the muscle and skin were sutured with absorbable and non-absorbable suture respectively. Iodine disinfectant was applied on the area to disinfect the skin after suturing. After surgery, the pigs were housed in recovery room for one week to allow recovery and then equally divided into three treatment groups. For minimizing animal suffering and distress, the animal behavior, such as movement, posture, breathing, and muscle rigidity, was observed every day. The 2 mg/kg of Carprofen was subcutaneously administered for 24 hr interval when animals showed potential signs associated with pain or distress.

### Bone loss induction

**Fig 1:**
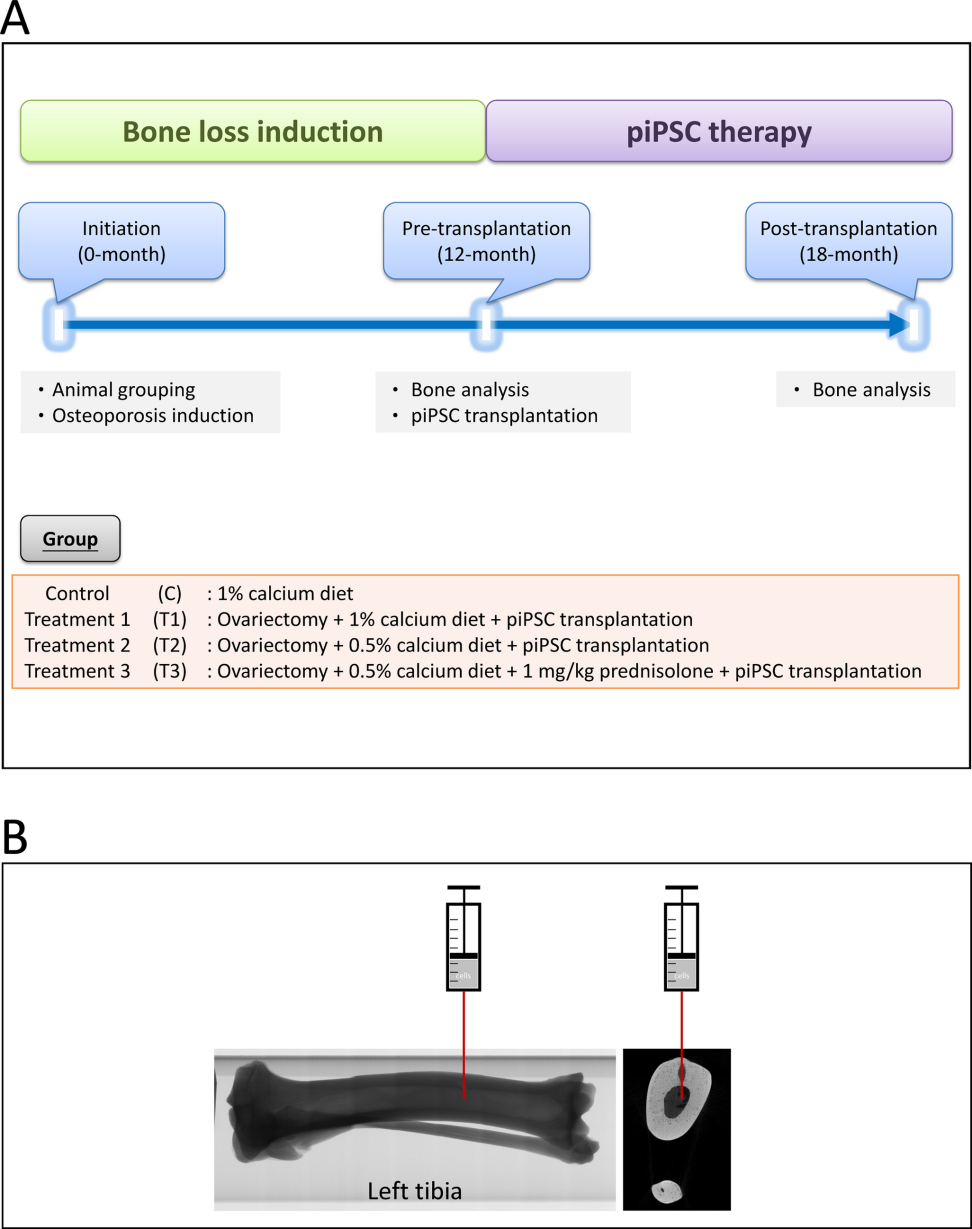
Study design of the experiment (A) and a schematic image for cell transplantation (B).

The ingredients of a standard diet (1% w/w calcium) and low calcium diet (0.5% w/w calcium) given in this study are shown in S1 Table. Application of prednisolone (Sigma-Aldrich) was filled in capsules and mixed with diets. Twenty female pigs were randomly and equally divided into four groups as follows: control (C): 1% calcium diet; treatment 1 (T1): ovariectomy + 1% calcium diet; treatment 2 (T2): ovariectomy + 0.5% calcium diet; treatment 3 (T3) ovariectomy + 0.5% calcium diet + 1 mg/kg of prednisolone. The pigs were weighed monthly, and two pigs in each group were sacrificed for bone loss evaluation after an induction period of twelve months. The rest of pigs in the treatment groups were subjected to cell transplantation and were subsequently maintained for another six months. The experimental design of this study is shown as Fig 1A.

### Cell transplantation

The pigs in T1, T2, and T3 group were anesthetized with 8 mg/kg body weight of Zoletil^®^ 50 (Virbac corporation), and the pre-operative preparation was performed as mentioned previously. To transplant piPSC-derived osteoblast-like cells, a transplanted hole in the left tibiae was exposed using a bone drill, and then 1×10^6^ piPSC-derived osteoblast-like cells in 10 μL of PBS were injected into the medullary cavity of left tibiae (Fig 1B). Left tibiae were subjected to cell transplantation, and right tibiae were maintained as internal controls. After cell transplantation, pigs were enabled free access to water and the same diets as mentioned previously (Fig 1A).

### Micro-CT scanning

The tibiae of pigs were obtained at the end of bone loss induction after six months of cell transplantation. After removal of muscle and adipose tissues, individual tibiae were immersed in 95% ethanol at 4°C for two days and later subjected to micro-CT scanning at an isotropic spatial resolution of 35 μm and a peak voltage of 100 kV using Bruker Skyscan 1076 *in vivo* micro-CT (Bruker, Kontich, Belgium). To determine microarchitectures of trabecular bone, including trabecular bone volume, thickness, separation, number, and total porosity, the region of interest (ROI) was selected at the center of tibia and separated from the cortical bone. Data for ROI from the proximal metaphysis to the distal metaphysis of tibiae [ROI (1)] and ROI at transplanted sites [ROI (2)] were analyzed using CT-Analyser software (Bruker). To measure the proportion of cortical bone in the cross-section of proximal metaphysis, proximal diaphysis, and distal diaphysis, ROI was selected at the periphery of tibia and separated from the trabecular bone. We calculated whole area of cross section (cortical region + trabecular region) and cortical region. The proportion of cortical bone was determined as cortical area/total area. Data were analyzed using ImageJ software (National Institutes of Health, USA).

### Biomechanical test

After micro-CT scanning, porcine tibiae were used to determine bone mechanical properties through three-point bending mechanical tests using MTS Bionix^®^ Servohydraulic Test System (MTS Systems Corporation, MN, USA) following the manufacturer's instruction. Individual tibiae were horizontally placed on rounded edges at a distance of 50 mm. A punch with a rounded notch was applied at the mid-shaft of diaphysis of the tibia at a speed of 1 mm/s. Force and deflection were automatically and continuously recorded until fracture. Maximum force (kgf), displacement (mm), stiffness (kgf/mm), and energy absorption until failure (kgf × mm) were also recorded.

### Mineral content analysis

All tibiae were oven-dried at 60°C for 24 h and mashed by a hammer and grinding machine. The contents of ash, calcium, and phosphorus were determined using Association of Official Analytical Chemists (AOAC) method 942.05, 968.08, and 965.17 [24], respectively.

### Statistical analysis

Data were analyzed by the analysis of variance using General Linear Model (GLM) procedure and Duncan’s multiple range test of Statistical Analysis System (SAS) [25]. Significant differences were determined as a P-value less than 0.05.

## Results

### piPSCs differentiated into osteoblast-like cells that express osteogenic markers

**Fig 2:**
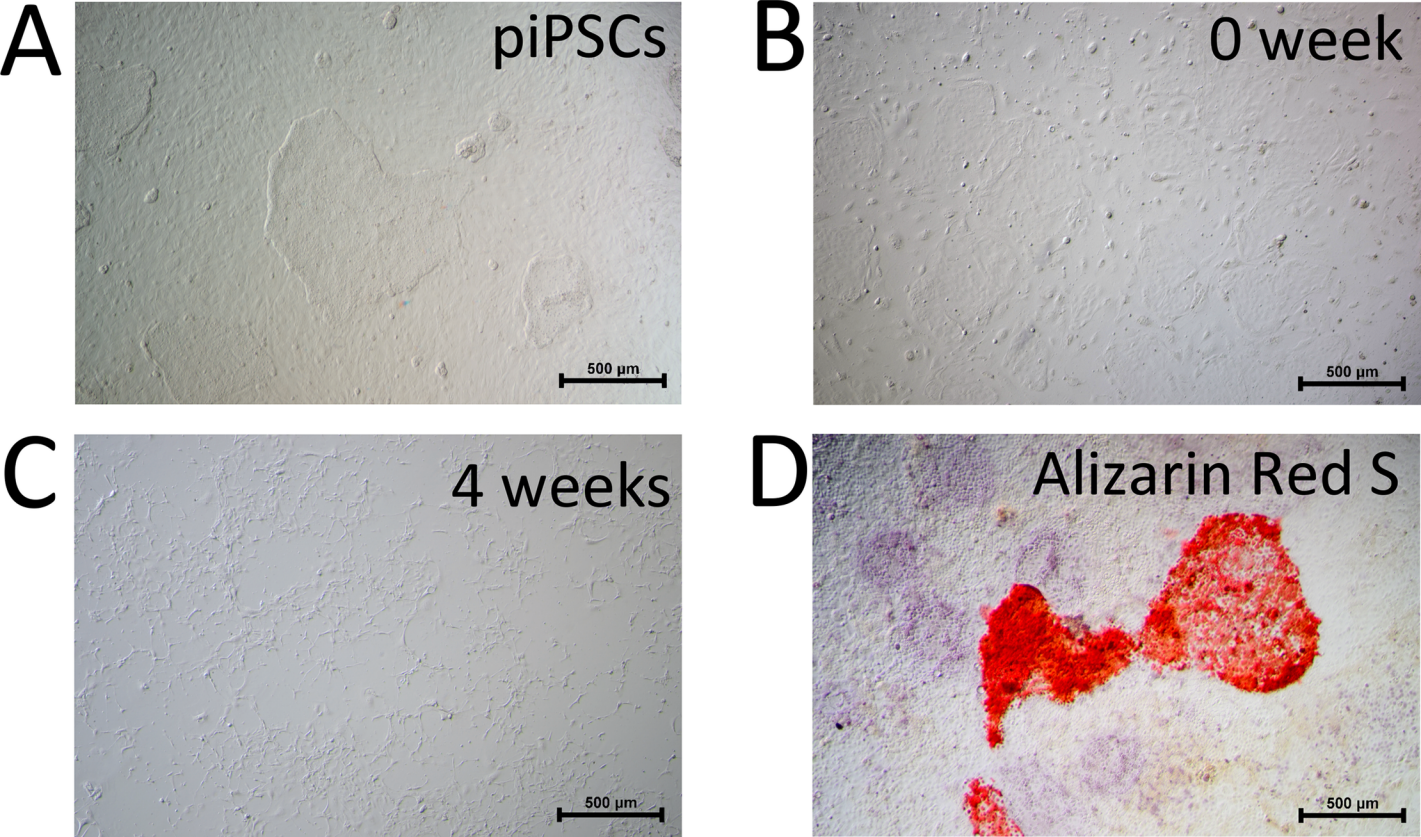
Differentiation of piPSC-derived osteoblast-like cells and expression of osteogenic markers. (A-C) Morphology of piPSCs before and after osteogenic induction. (D) Alizarin red S staining of piPSC-derived osteoblast-like cells after 4-week induction culture.

**Fig 3:**
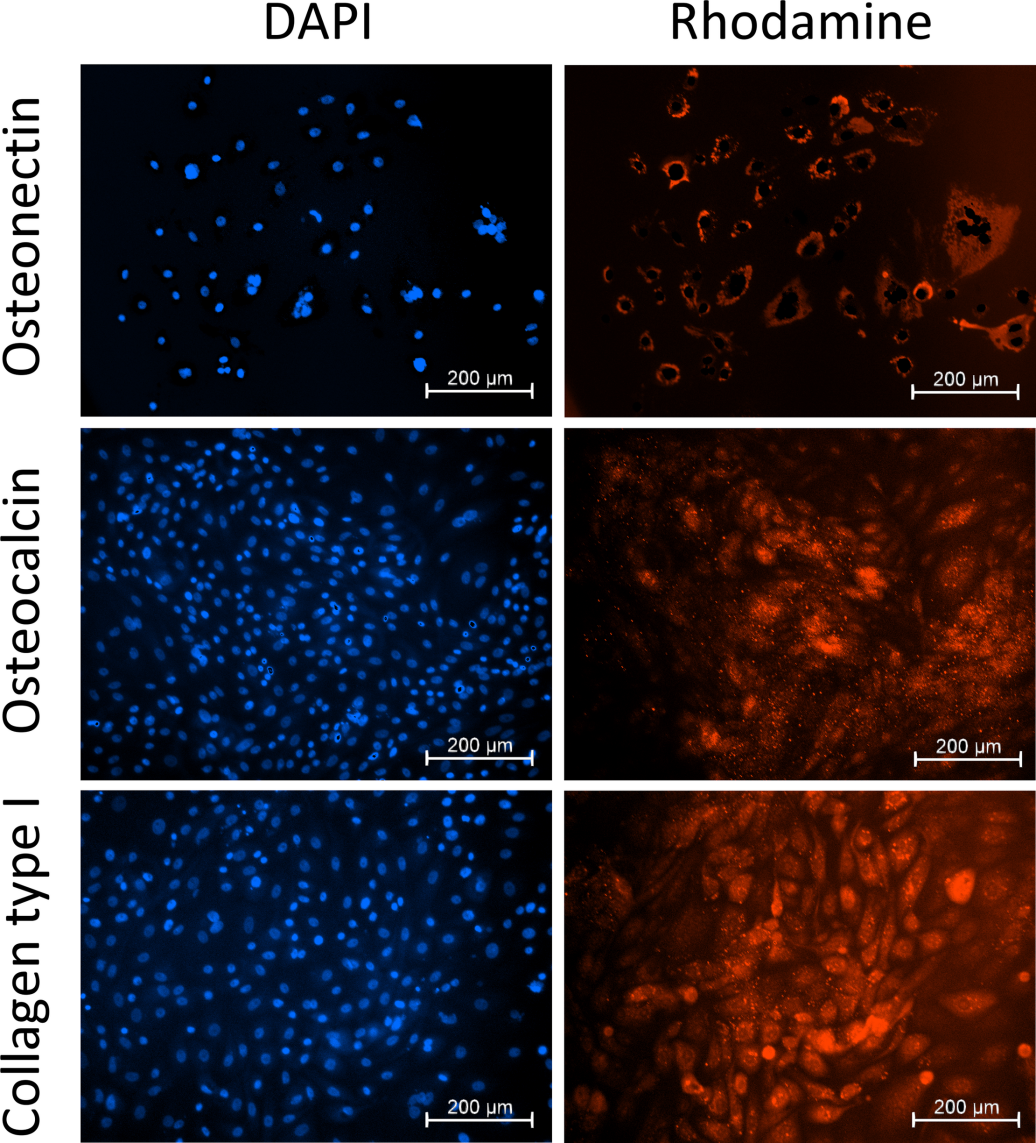
Expression of osteogenic markers of piPSC-derived osteoblast-like cells.

Before induction of osteogenic differentiation, piPSCs showed a typical ESC-like morphology (Fig 2A). To induce differentiation of osteoblast-like cells, trypsinized piPSCs were maintained in osteogenic medium, containing MEM-α, 10% FBS, 50 μM L-ascorbic acid, 10 mM β-glycerophosphate, 100 nM dexamethasone, 10 nM 1α,25(OH)2D3, and 40 ng/mL BMP2. After 4 weeks of culture, cell morphology changed from oval or round shape to spindle-like shape (Fig 2B and 2C). Alizarin red S staining was positive, suggesting that calcium and phosphate were deposited in piPSC-derived osteoblast-like cells (Fig 2D). Furthermore, expressions of osteogenic markers, including osteonectin, osteocalcin, and collagen type I, were confirmed by immunocytochemical staining (Fig 3).

### Growth retardation in prednisolone-treated Lanyu pigs

During the twelve months of the experimental period, the body weight of Lanyu pigs in all groups was monitored monthly. The linear relationship between body weight and the experimental period was time-dependent. After three months of bone loss induction, the average body weight of pigs in the control, T1, T2 and T3 groups was 60.6 ± 4.3 kg, 56.4 ± 5.4 kg, 55.6 ± 5.0 kg, and 48.4 ± 3.2 kg, respectively. At the end of the experiments, average body weight in the control, T1, T2 and T3 groups was 101.0 ± 10.9 kg, 92.6 ± 12.6 kg, 92.2 ± 8.6 kg, and 65.7 ± 10.5 kg, respectively (S1A Fig). The effect of ovariectomy, 0.5% calcium diet, and prednisolone treatment on growth retardation was noted, and the average body weight in the T3 group was significantly lower than that in the control group after three months of bone loss induction (P < 0.05). Conversely, there was no significant difference in body weight found between T1 and T2 group, suggesting that calcium content in diets used in the present study might not affect their body weight gain. Furthermore, low body weight was related to the length of the tibia (S1B and S1C Fig). At the end of the bone loss induction period, the tibia length in the control, T1, T2, and T3 groups was 168.6 ± 2.4 mm, 168.5 ± 5.1 mm, 174.1 ± 6.0 mm, and 150.4 ± 7.1 mm, respectively (S1C Fig). The bone loss induction treatment combined with ovariectomy, 0.5% calcium diet, and prednisolone application for pigs in the T3 group significantly shortened their tibia length (P < 0.05).

### Prednisolone treatment promotes trabecular and cortical bone loss

**Fig 4:**
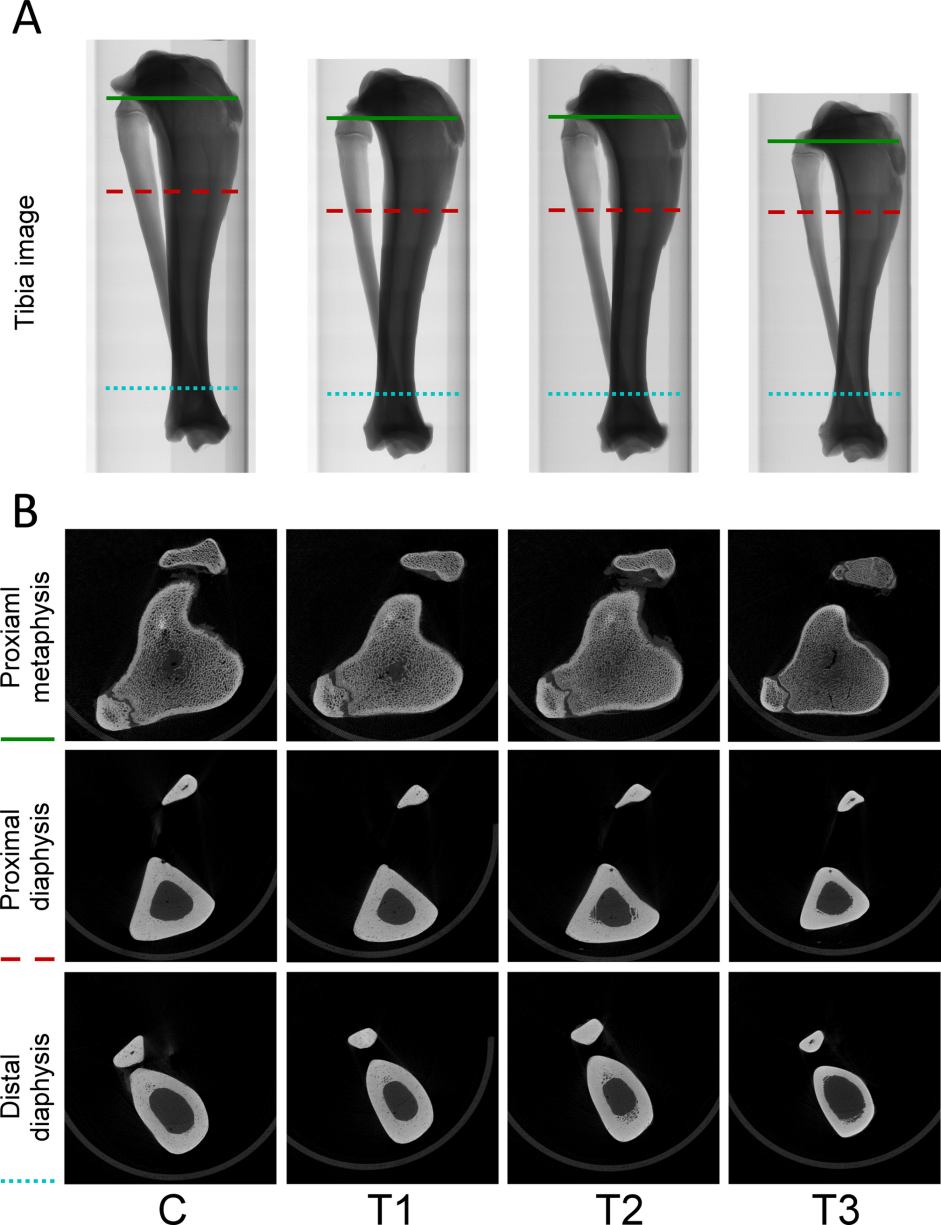
Poor bone structures were evident in the T3 group. (A) Representative micro-CT image of tibiae after twelve months of bone loss induction. (B) Representative micro-CT image of proximal metaphysis, proximal diaphysis, and distal diaphysis retrieved from green solid line, red dashed line, and blue dotted line, respectively. C, control; T1, treatment 1; T2, treatment 2; T3, treatment 3.

**Fig 5:**
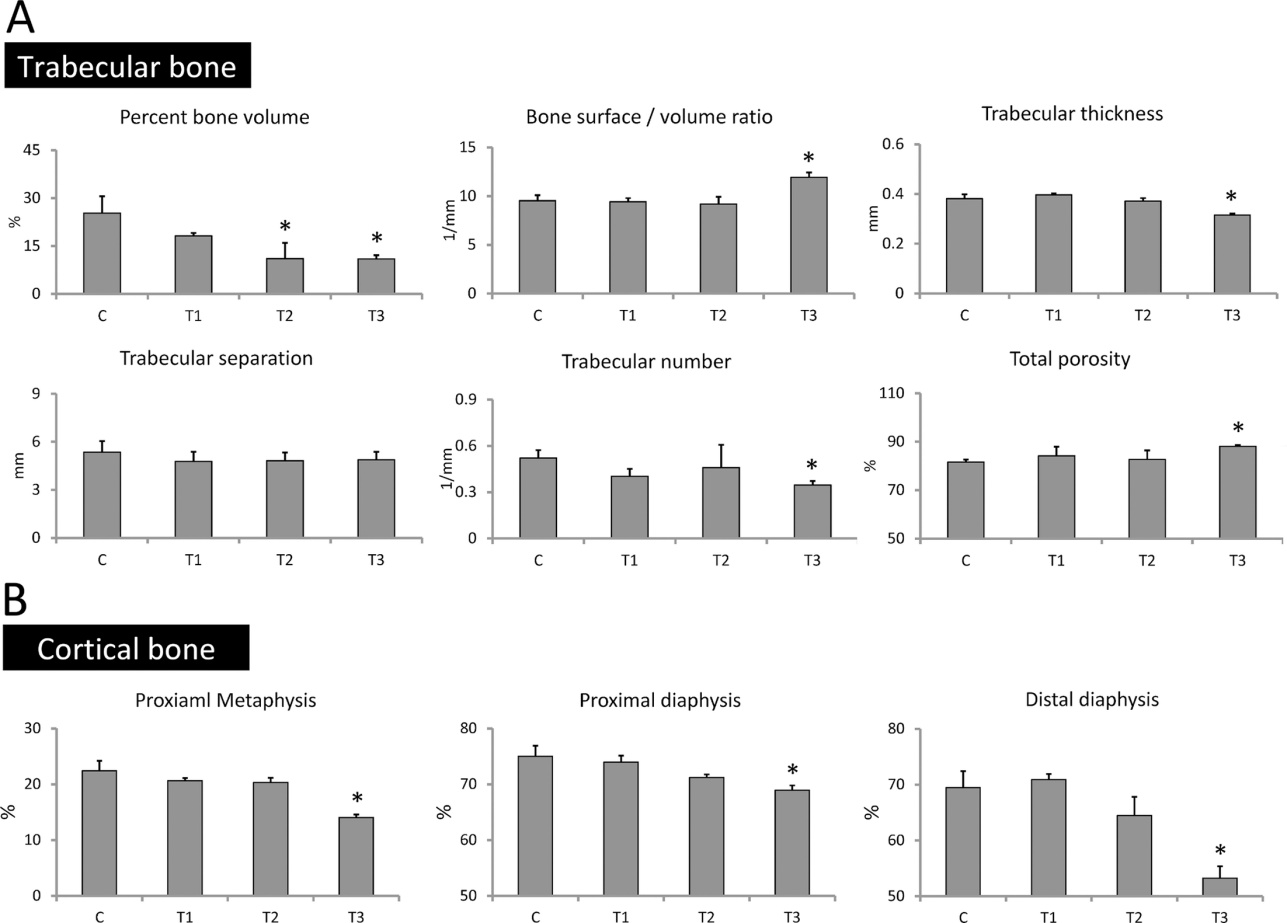
Reduced trabecular and cortical bone structural parameters in the T3 group. (A) Quantification of trabecular bone structural parameters. (B) Quantification of cortical bone structural parameters. C, control; T1, treatment 1; T2, treatment 2; T3, treatment 3. *: P < 0.05 versus C (Duncan’s multiple range test).

Micro-CT analysis was performed at the end of bone loss induction to investigate whether the treatments we applied could promote bone loss in tibiae. For trabecular bone analysis, ROI from the proximal metaphysis to the distal metaphysis was measured. Again, tibia images confirmed that the tibia length of pigs in the T3 group was the shortest (Fig 4A). At the sites of proximal tibial metaphysis, micro-CT images showed that the trabecular bone of pigs in the T1 and T2 group exhibited dense and compact structures, similar to that in the control group. However, the images of loose trabecular bone found in the T3 group suggest that prednisolone treatment might induce progression of bone loss (Fig 4B). Furthermore, the percent bone volume, bone surface/volume ratio, trabecular thickness, trabecular separation, trabecular number, and total porosity of trabecular bone were examined to verify the progression of bone loss. Results showed that the percent bone volume in the control, T1, T2, and T3 groups was 25.34 ± 5.21%, 18.16 ± 0.89%, 11.07 ± 4.88%, and 10.96 ± 1.16%, respectively. The level of bone surface/volume ratio in the control, T1, T2, and T3 groups was 9.53 ± 0.56 1/mm, 9.42 ± 0.37 1/mm, 9.20 ± 0.71 1/mm, and 11.92 ± 0.51 1/mm, respectively. The level of trabecular thickness in the control, T1, T2, and T3 groups was 0.38 ± 0.02, 0.40 ± 0.01, 0.37 ± 0.01, and 0.32 ± 0.01 mm, respectively. The level of trabecular separation in the control, T1, T2, and T3 groups was 5.35 ± 0.69 mm, 4.77 ± 0.60 mm, 4.82 ± 0.50 mm, and 4.87 ± 0.50 mm, respectively. Trabecular numbers in the control, T1, T2, and T3 groups was 0.52 ± 0.06 1/mm, 0.40 ± 0.09 1/mm, 0.46 ± 0.02 1/mm, and 0.35 ± 0.09 1/mm, respectively. The level of total porosity in the control, T1, T2, and T3 groups was 81.61 ± 1.80%, 84.20 ± 2.44%, 82.65 ± 1.38%, and 88.07 ± 1.27%, respectively (Fig 5A). These results reveal that treatment combining ovarietomy and 0.5% calcium diet in the T2 group reduced the percent bone volume when compared with that in the control group, but other parameters in the T2 group remained at the same level as in the control group. Although there were no significant differences found among trabecular separation in all groups, other parameters detected in the T3 group were significantly different from those in the other groups. These results indicate that the treatment combining ovariectomy, 0.5% calcium diet, and prednisolone application efficiently promotes trabecular bone loss in tibiae of Lanyu pigs. For cortical bone analysis, the proximal metaphysis, proximal diaphysis, and distal diaphysis were selected for micro-CT scanning. The boundary between trabecular and cortical bone was difficult to identify by micro-CT images at the sites of proximal tibial metaphysis in the control, T1, and T2 groups. However, in the T3 group, this boundary was clear, and cortical bone thickness was also attenuated (Fig 4B). At the site of proximal and distal diaphysis, the cortical bone thickness in the control, T1, and T2 groups was thicker than that in the T3 group (Fig 4B). Next, we analyzed micro-CT images using ImageJ software to determine the proportion of cortical bone in the regions of proximal metaphysis, proximal diaphysis, and distal diaphysis. Results showed that the proportion at the site of proximal metaphysis in the control, T1, T2, and T3 groups was 22.41 ± 1.78%, 20.64 ± 0.45%, 20.33 ± 0.82%, and 14.06 ± 0.51%, respectively. The proportion at the site of proximal diaphysis in the control, T1, T2, and T3 groups was 75.03 ± 1.87%, 73.96 ± 1.17%, 71.21 ± 0.54%, and 68.94 ± 0.84%, respectively. The proportion at the site of distal diaphysis in the control, T1, T2, and T3 groups was 69.47 ± 2.91%, 70.91 ± 0.97%, 64.47 ± 3.32%, and 53.25 ± 2.10%, respectively (Fig 5B). The proportions of cortical bone in selected regions were significantly lower in the T3 group (P < 0.05). Approximately 23%, 8%, and 37% reduction of the proportions of cortical bone was found in the regions of proximal metaphysis, proximal diaphysis, and distal diaphysis, respectively. These results suggest that combining treatments of ovariectomy, 0.5% calcium diet, and prednisolone application not only impaired trabecular bone microarchitectures but also promoted cortical bone loss in tibiae.

### Dietary calcium restriction and prednisolone application impaired bone strength in ovariectomized Lanyu pigs

After micro-CT scanning, the tibiae were subjected to bone strength tests using a three-point bending test. Individual tibiae were horizontally placed on edges, and a punch was applied at the mid-shaft of the tibia diaphysis (S2A Fig). Results showed that maximum force in the control, T1, T2, and T3 groups was 830.84 ± 65.48 kgf, 744.27 ± 37.80 kgf, 700.83 ± 28.14 kgf, and 430.33 ± 29.99 kgf, respectively. Displacement in the control, T1, T2, and T3 groups was 2.61 ± 0.13 mm, 2.51 ± 0.21 mm, 2.56 ± 0.32 mm, and 1.79 ± 0.12 mm, respectively. Stiffness in the control, T1, T2, and T3 groups was 322.74 ± 43.19 kgf/mm, 297.71 ± 10.23 kgf/mm, 275.67 ± 19.30 kgf/mm, and 239.73 ± 2.92 kgf/mm, respectively. Energy absorption in the control, T1, T2, and T3 groups was 2150.92 ± 55.70 kgf × mm, 1886.72 ± 252.84 kgf × mm, 1803.87 ± 325.40 kgf × mm, and 779.19 ± 103.30 kgf × mm, respectively (S2B–S2E Fig). Maximum force was significantly attenuated in the T2 and T3 groups, suggesting that dietary calcium restriction significantly impaired bone strength in ovariectomized Lanyu pigs (P < 0.05), and prednisolone treatment further accelerated the progression of decreased bone strength (P < 0.05). However, significant reduction of other parameters, including displacement, stiffness, and energy absorption, were only found in the T3 group, which was treated with ovariectomy, 0.5% calcium diet, and prednisolone (P < 0.05). After bone loss induction, the level of maximum force, displacement, stiffness, and energy absorption in the T3 group was attenuated by 48%, 31%, 26%, and 64%, respectively.

### Application of prednisolone reduced bone mineralization

The mineral content of individual tibiae, including ash, calcium, and phosphorus, were determined. In the control, T1, T2, and T3 groups, ash content was 52.21 ± 0.18%, 51.88 ± 0.27%, 51.95 ± 0.67%, and 46.01 ± 1.41%, respectively. Calcium content was 20.52 ± 0.17%, 21.02 ± 0.03%, 20.70 ± 0.42%, and 17.82 ± 0.20%, respectively, and phosphorus content was 8.20 ± 0.28%, 8.52 ± 0.10%, 8.41 ± 0.14%, and 7.12 ± 0.13%, respectively (S3 Fig). Bone mineral content in the T1 and T2 groups remained the same as that in the control group, and only Lanyu pigs in the T3 group with prednisolone application exhibited significantly lower levels of ash, calcium, and phosphorus content (P < 0.05), which was reduced by approximately 11%, 13%, and 13%, respectively.

### Transplanted piPSC-derived osteoblast-like cells maintained trabecular bone structures from the proximal metaphysis to the distal metaphysis in tibiae

**Fig 6:**
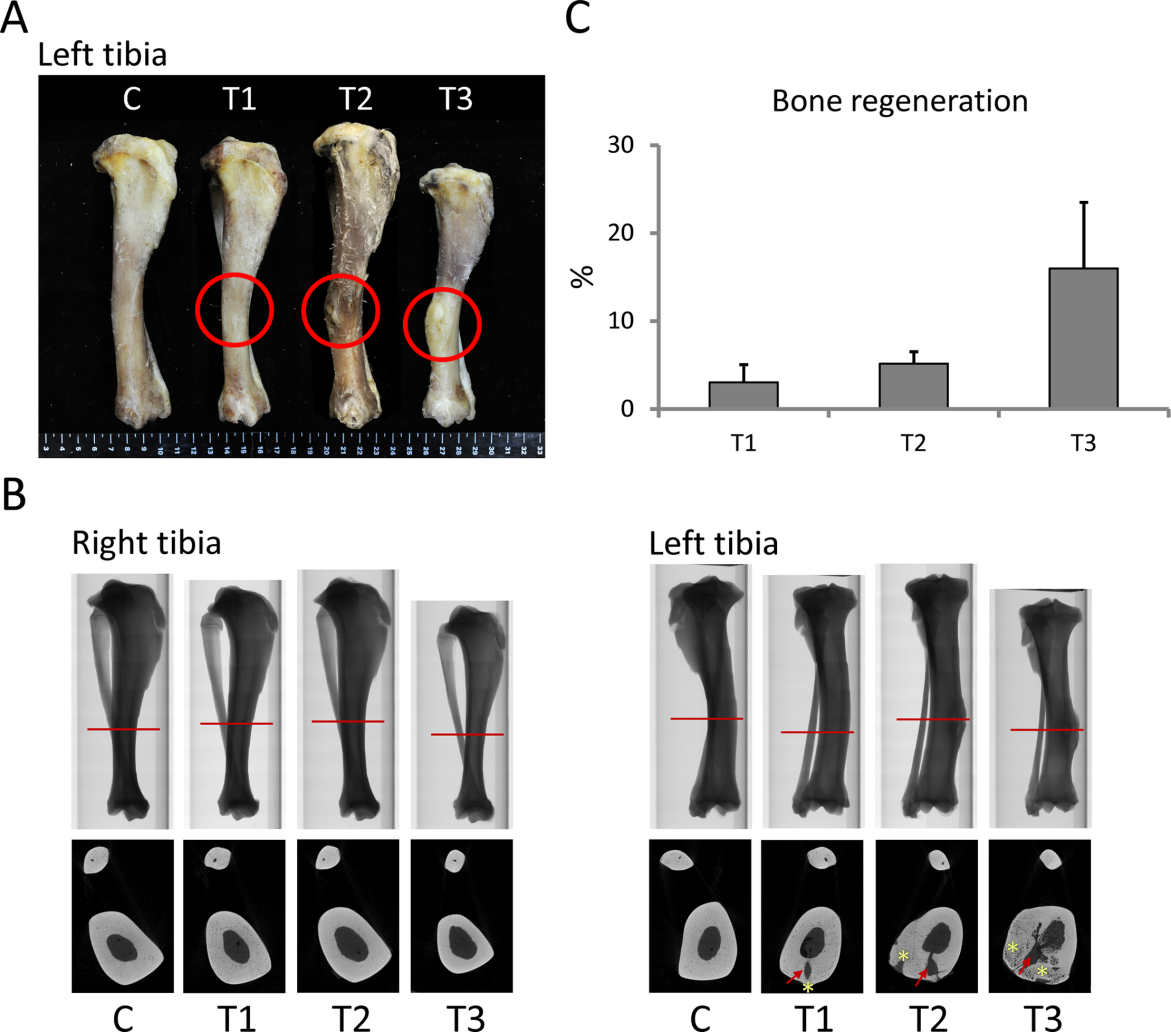
Bone regeneration was evidenced at the transplanted site. (A) Image of tibiae after cell transplantation (red circles indicated transplanted sites). (B) Micro-CT images illustrating transplanted sites retrieved from sections indicated by red lines (red arrows indicated a hole exposed by bone drill; yellow stars indicated regenerated bone). (C) The rate of bone regeneration in each treatment group. C, control; T1, treatment 1; T2, treatment 2; T3, treatment 3. N = 3 in each group.

**Fig 7:**
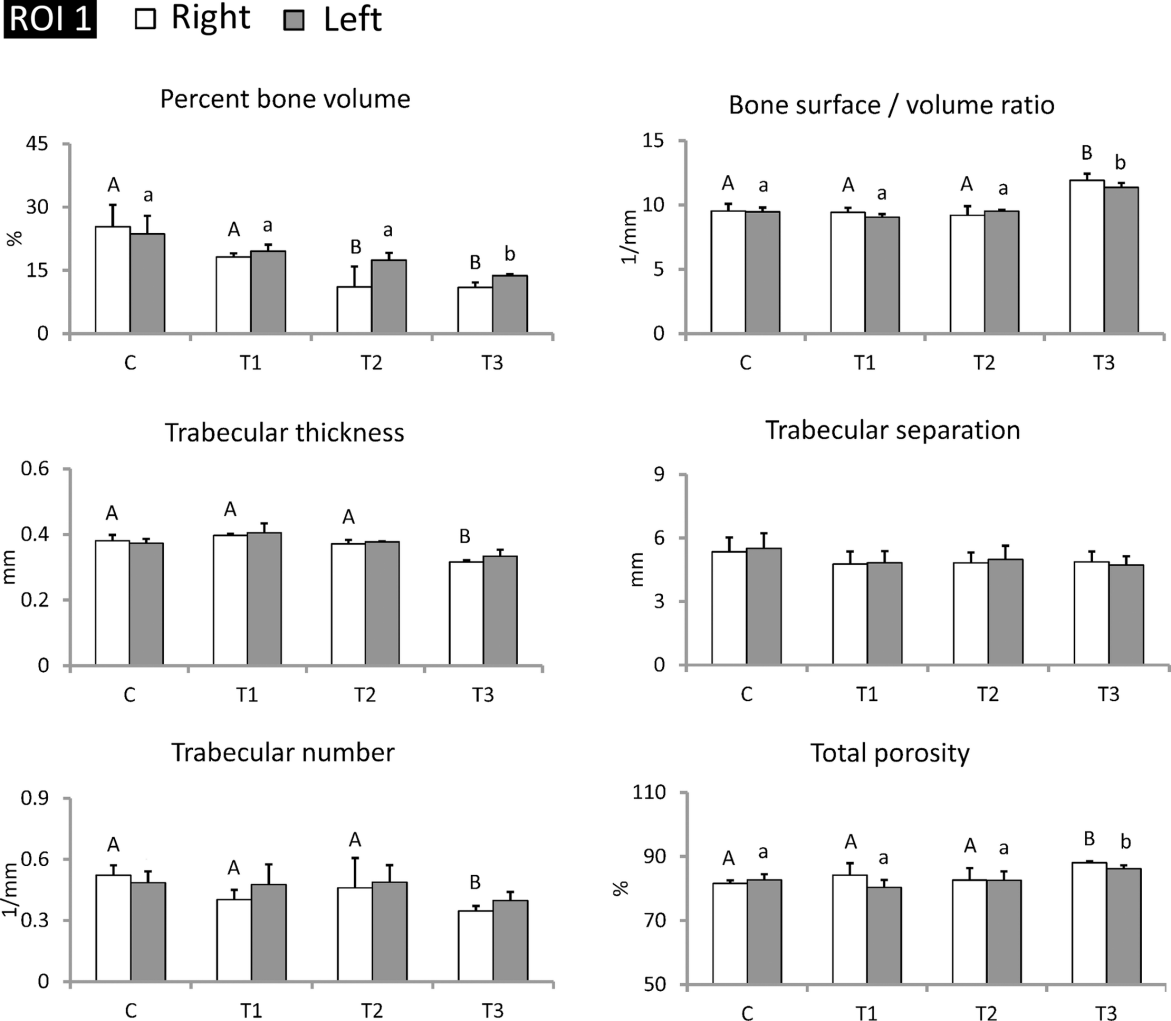
Transplantation of piPSC-derived osteoblast-like cells improved trabecular thickness and number in the left tibiae of the T3 group. The range of ROI (1) was selected from the proximal metaphysis to the distal metaphysis in tibia. Left tibiae were subjected to cell transplantation, and right tibiae were maintained as internal control. C, control; T1, treatment 1; T2, treatment 2; T3, treatment 3. ^AB/ab^: Values in the same site with different letters indicate significant differences (Duncan’s multiple range test). N = 3 in each group.

After twelve months of bone loss induction, left tibiae were subjected to cell transplantation, and right tibiae were maintained as internal controls. After cell transplantation, pigs were maintained for another six months and later sacrificed for tibiae collection. Tibiae were examined using micro-CT analysis to determine whether transplanted piPSC-derived osteoblast-like cells improved trabecular bone mass. Results showed that the transplanted sites of treatment groups exhibited bone regeneration, and regenerated structures at transplanted sites clearly presented in micro-CT images (Fig 6A and 6B). Among these groups, the T3 group exhibited the most obvious bone regeneration. Rate of bone regeneration in the T1, T2, and T3 groups was 3.04 ± 1.98%, 2.32 ± 1.34%, and 15.97 ± 7.51%, respectively (Fig 6C). Next, we selected ROI (1) (from the proximal metaphysis to the distal metaphysis of tibia) and employed micro-CT for evaluating trabecular bone parameters. Results revealed that trabecular thickness of the left tibiae in control, T1, T2, and T3 groups was 0.37 ± 0.01 mm, 0.40 ± 0.02 mm, 0.38 ± 0.01 mm, and 0.35 ± 0.02 mm, respectively. Trabecular number of the left tibiae in control, T1, T2, and T3 groups was 0.49 ± 0.04 1/mm, 0.48 ± 0.02 1/mm, 0.49 ± 0.05 1/mm, and 0.40 ± 0.03 1/mm, respectively. Compared with the control group, these two parameters in the right tibiae of the T3 group exhibited significantly lower parameters (P < 0.05), but those in the left tibiae of the T3 group were improved. These results demonstrate that trabecular thickness and number in the left tibiae of the T3 group were recovered to the same level as seen in the control group by transplanting piPSC-derived osteoblast-like cells (Fig 7).

### Transplanted piPSC-derived osteoblast-like cells maintained trabecular bone structures at transplanted sites

**Fig 8:**
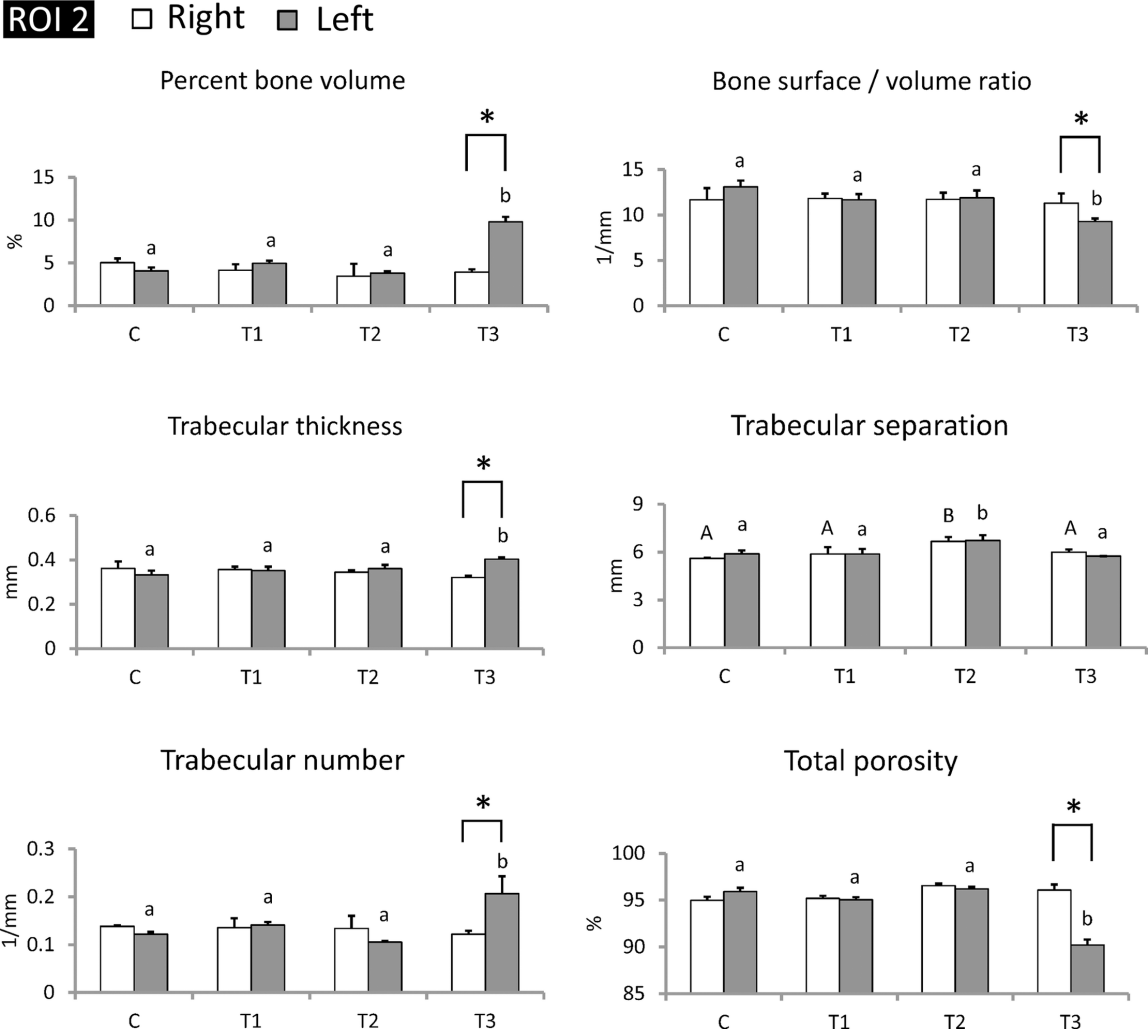
piPSC-derived osteoblast-like cells markedly improved trabecular microarchitecture in the left tibiae of T3 group at transplanted sites. Transplanted cells ameliorated trabecular bone volume, thickness, separation, number, and total porosity at transplanted sites in the T3 group. The range of ROI (2) at transplanted sites. Left tibiae were subjected to cell transplantation, and right tibiae were maintained as internal control. C, control; T1, treatment 1; T2, treatment 2; T3, treatment 3. ^AB/ab^: Values in the same site with different letters indicate significant differences. *: P < 0.05 versus Right (Duncan’s multiple range test). N = 3 in each group.

To further confirm the therapeutic effects of piPSC-derived osteoblast-like cells at transplanted sites, micro-CT analysis focused on ROI (2) (at transplanted sites). Except for trabecular separation, almost all selected trabecular bone parameters in the right tibiae for all groups remained at the same level, with left tibiae transplanted piPSC-derived osteoblast-like cells exhibiting significant improvements in trabecular bone volume, thickness, number, and total porosity (Fig 8) (P < 0.05). Percent bone volume in the control, T1, T2, and T3 groups was 4.1 ± 0.4%, 5.0 ± 0.3%, 3.8 ± 0.2%, and 9.8 ± 0.6%, respectively. Bone surface/volume ratio in the control, T1, T2, and T3 groups was 13.01 ± 0.7 1 /mm, 11.7 ± 0.6 1/mm, 11.9 ± 0.8 1/mm, and 9.3 ± 0.6 1/mm, respectively (P < 0.05). Trabecular thickness in the control, T1, T2, and T3 groups was 0.33 ± 0.02 mm, 0.35 ± 0.02 mm, 0.36 ± 0.02 mm, and 0.40 ± 0.01 mm, respectively (P < 0.05). Trabecular number in the control, T1, T2, and T3 groups was 0.12 ± 0.01 1/mm, 0.14 ± 0.01 1/mm, 0.11 ± 0.01 1/mm, and 0.21 ± 0.04 1/mm, respectively (P < 0.05). Total porosity in the control, T1, T2, and T3 groups was 95.9 ± 0.40%, 95.0 ± 0.29%, 96.2 ± 0.22%, and 90.2 ± 0.6%, respectively (P < 0.05) (Fig 8). Trabecular bone parameters in the left tibiae of T3 group were not only greater than those in the right tibiae of T3 group but were also higher than that in the control group (Fig 8). These results indicate that piPSC-derived osteoblast-like cells remarkably promoted development of trabecular bone mass in the medullary cavity of left tibiae, suggesting that piPSC-derived osteoblast-like cells might ameliorate the progression of trabecular bone loss.

### Transplanted piPSC-derived osteoblast-like cells maintained cortical bone structures in the proximal metaphysis

**Fig 9:**
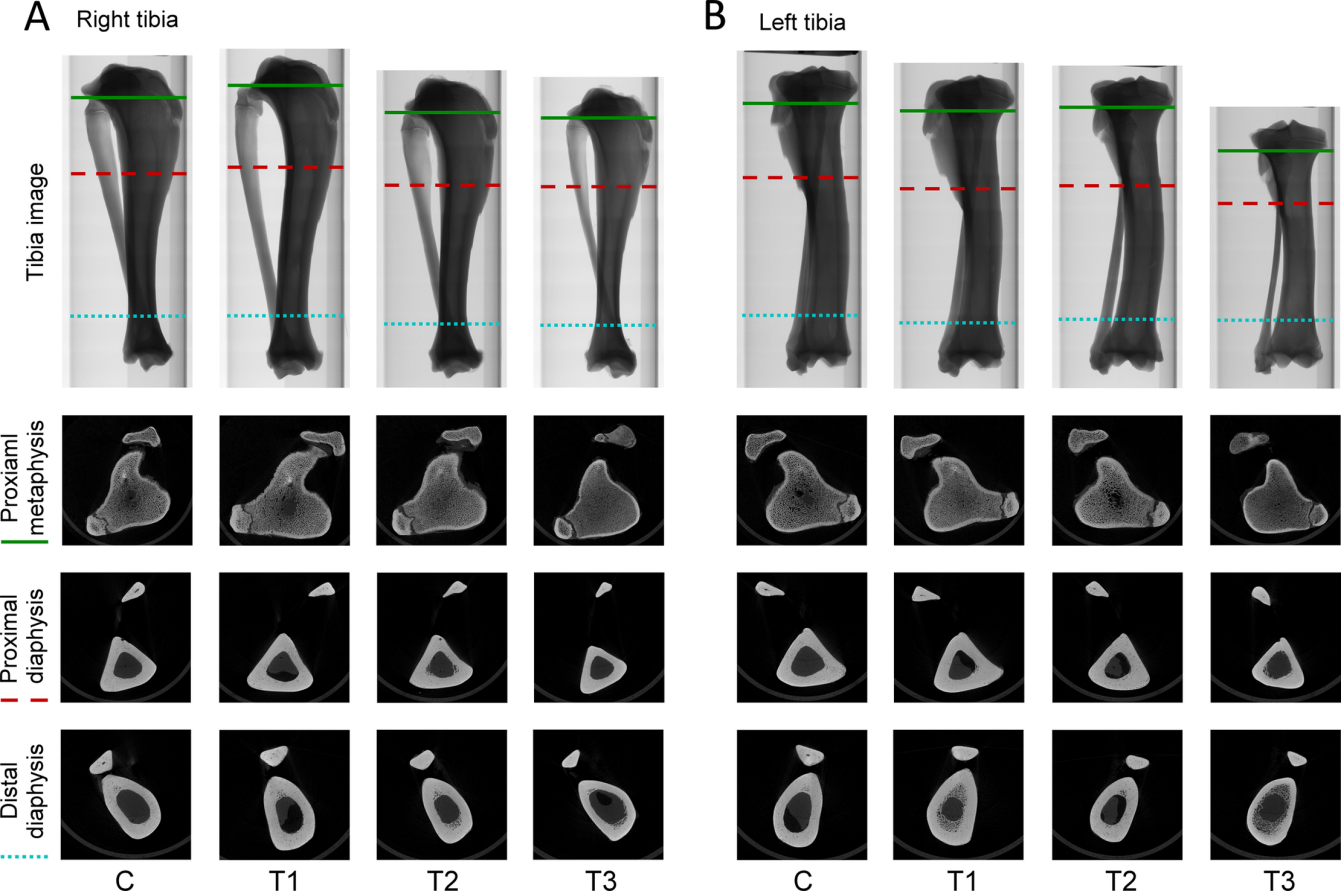
Improvement of cortical bone structures in the T3 group. (A) Representative micro-CT image of tibiae after cell transplantation. (B) Representative micro-CT image of proximal metaphysis, proximal diaphysis, and distal diaphysis retrieved from green solid line, red dashed line, and blue dotted line, respectively. C, control; T1, treatment 1; T2, treatment 2; T3, treatment 3. N = 3 in each group.

**Fig 10:**
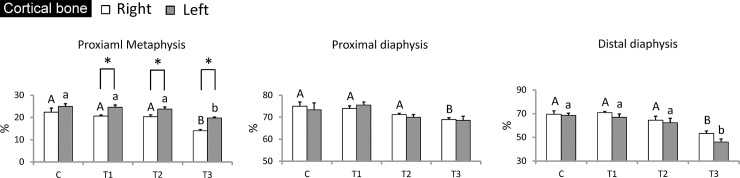
piPSC-derived osteoblast-like cells markedly improved cortical microarchitecture in proximal metaphysis of the left tibiae in the T3 group. Proximal metaphysis, proximal diaphysis, and distal diaphysis were examined, and only proximal metaphysis showed significant improvement. C, control; T1, treatment 1; T2, treatment 2; T3, treatment 3. ^AB/ab^: Values in the same site with different letters indicate significant differences. *: P < 0.05 versus Right (Duncan’s multiple range test). N = 3 in each group.

After cell transplantation, we also determined the proportion of cortical bone in selected regions of tibiae. For cortical bone analysis, proximal metaphysis, proximal diaphysis, and distal diaphysis were examined using micro-CT scans. At sites of proximal metaphysis, the boundary between trabecular and cortical bone in the left tibiae was still as difficult to identify as that in the right tibiae (Fig 9). At the site of proximal and distal diaphysis, the cortical bone thickness in the control, T1, and T2 groups was also thicker than that in the T3 group (Fig 9). Results of the proportion of cortical bone in the left tibiae showed that the proportion at the site of proximal metaphysis in the control, T1, T2, and T3 groups was 24.94 ± 1.21%, 24.63 ± 0.95%, 23.80 ± 0.93%, and 19.76 ± 0.42%, respectively. The proportion at the site of proximal diaphysis in the control, T1, T2, and T3 groups was 73.38 ± 3.12%, 75.54 ± 1.37%, 70.00 ± 1.18%, and 68.57 ± 1.88%, respectively. The proportion at the site of distal diaphysis in the control, T1, T2, and T3 groups was 68.52 ± 1.85%, 66.94 ± 2.72%, 62.39 ± 3.64%, and 46.10 ± 2.54%, respectively (Fig 10). Interestingly, the proportion of cortical bone in the proximal metaphysis of left tibiae of treatment groups was significantly higher than that of right tibiae of treatment groups (P < 0.05), although this improvement in the left tibiae of the T3 group was still not sufficient to recover the proportion of cortical bone to levels as high as that in the control group. These results suggest that piPSC-derived osteoblast-like cells might promote the development of cortical bone in the proximal metaphysis.

## Discussion

Ovariectomy has been used for establishment of animal models of osteoporosis in several animals, such as rats, rabbits, goats, sheep, and miniature pigs [1,15,26,27]. Long-term use of glucocorticoids also results in bone loss and low bone turnover [13–15]. The underlying mechanisms that lead to glucocorticoid-induced bone loss include reduced osteoblast and osteocyte lifespan, promotion of osteoclast survival, and reduced renal reabsorption of calcium [28]. However, selection of a suitable animal model for osteoporosis study is difficult [29]. Rodgers et al. (1993) [30] stated that convenience, relevance (comparability to the human condition), and appropriateness (a complex of other factors that make a given species the best for studying a particular phenomenon) were the three key points when discussing possible models for osteoporosis research. Because bone structure in mini pigs is similar to that in humans [30,31], mini pigs have become a popular animal model for osteoporosis research. Furthermore, a previous study demonstrated that ovariectomy and calcium-restricted diet induces loss of bone mass, trabecular microarchitectures, and bone strength in Sinclair S1 mini pigs [5]. In addition, glucocorticoid treatment in Göttingen mini pigs was reported to result in bone loss and low bone mineral density [16], while Ibandronate, a potent bisphosphonate drug, reversed the loss of bone mineral density and strength [32]. Therefore, mini pigs might be a suitable model for osteoporosis research and for nonclinical evaluation of therapeutic effects on bone loss, trabecular microarchitectures, and bone strength [1,31]. In the present study, we attempted to promote bone loss in Lanyu pigs using ovariectomy, calcium-restricted diet, and glucocorticoid treatment. We further investigated whether piPSC-derived osteoblast like-cells exert therapeutic effects on bone mass improvements in Lanyu pigs.

Ovariectomy in mini pigs results in low blood osteocalcin levels, high blood parathyroid hormone levels, and decreased bone mineral density [1]. Calcium plays an important role in regulating bone remodeling and bone mass [10]. Low dietary calcium treatment in pigs reduces calcium retention, bone mass, and bone mineral content [33]. In our study, reduction of trabecular bone volume was found in ovariectomized Lanyu pigs fed a calcium-restricted diet (T2 group) (Fig 4A).

Long-term administration of glucocorticoids results in bone loss [13–15]. Furthermore, an inhibitory effect on body weight, food intake, and appetite peptide expression have been confirmed in rodents [34,35]. In previous studies, no effect on body weight from glucocorticoids administration was reported in growing mini pigs, presumably because the rapid growing stage was superimposed on the effect of glucocorticoids [36]. However, in the present study, long-term prednisolone treatment in Lanyu pigs did have an inhibitory effect on body weight (S1A Fig) and induced growth retardation with shorter tibia length (S1B and S1C Fig and Fig 3A). This effect might be the reason that Lanyu pigs used in this study are 6 months old, which have already reached the age of puberty for this miniature breed.

Basic morphometric indices of trabecular bone used in the present study included the measurement of percent bone volume [bone volume (BV) / total volume of interest (TV)], bone surface/volume ratio, trabecular thickness, trabecular separation, trabecular number, and total porosity. Percent bone volume is used to evaluate relative changes in bone volume. The bone surface/volume ratio is defined as the ratio of segmented bone surface to segmented bone volume. Trabecular thickness, trabecular separation, and trabecular number indicate the mean thickness of the trabeculae, the mean distance between the trabeculae, and the average number of trabeculae per unit length, respectively. Total porosity is the total volume of open and closed pores [37,38]. Our results showed that Lanyu pigs treated with prednisolone exhibited shorter bone length and poor trabecular bone structures. These results were similar to a previous study in female Göttingen mini pigs, which exhibited short femur length and low trabecular thickness in L2 vertebrae after subcutaneous injection five days a week with 0.5 mg/kg/day of prednisolone for 26 weeks [36]. In this study, all deficiencies in bone microarchitecture (Figs 3 and 4), lower bone strength (S2 Fig), and less bone mineral content (S3 Fig) were found in Lanyu pigs induced by combination of ovariectomy, 0.5% calcium diet, and prednisolone treatment in the T3 group. Hence, individual treatment or combination of ovariectomy and calcium restriction might be not sufficient, and prednisolone administration should also be applied to properly induce osteoporosis in Lanyu pigs.

Recently, stem cell therapy for bone loss has been confirmed to be effective via local or systemic cell transplantation into osteoporotic animal models [9]. Previous studies demonstrated that MSCs systemically transplanted into mice markedly improved bone microarchitectural characteristics and induced osteoblastogenesis in long bone. These achievements demonstrated that MSC transplantation may be a treatment option to recover bone density in osteoporosis [39,40]. However, very limited literature has documented use of ESCs and iPSCs for osteoporosis therapy [41,42]. Because of the difficulty of osteogenic lineage differentiation, references concerning differentiation of iPSC-derived osteoblast-like cells are also scarce [9]. Our study confirmed that piPSCs could differentiate into osteoblast-like cells (Fig 2). Furthermore, we also confirmed that local transplantation of piPSC-derived osteoblast-like cells can ameliorate trabecular and cortical bone structures in tibiae of Lanyu pigs with corresponding improvements in trabecular bone volume, thickness, separation, number, and total porosity (Figs 5–9). These achievements for piPSC-derived osteoblast-like cell therapy are also uncovered in the rat models of osteoporosis [43]. We believe that these new evidences showed in the present study could enhance the application of piPSCs on regenerative medicine in the future. In conclusion, combining treatments of ovariectomy, low calcium diet, and prednisolone are an effective approach to promote trabecular and cortical bone loss in Lanyu pigs. The therapeutic potential of piPSC-derived osteoblast-like cells was also confirmed via cell transplantation into the left tibiae of Lanyu pigs. These findings reveal the therapeutic potential of piPSCs for glucocorticoid-induced bone loss in pig models.

## Supporting information

S1 Table Ingredients of experimental diets. (DOCX)

S1 Fig Growth retardation in the T3 group. (A) Weight of Lanyu pigs during the experimental period. (B) Representative image of tibiae after twelve months of bone loss induction. (C) Results of tibia length after twelve months of bone loss induction. C, control; T1, treatment 1; T2, treatment 2; T3, treatment 3. *: P < 0.05 versus C (Duncan’s multiple range test).
(TIF)

S2 Fig Low bone strength in the T3 group. (A) Representative image of tibia on the mechanical test equipment while three-point bending test. (B-E) Quantification of displacement, maximum force, stiffness, and energy parameters. C, control; T1, treatment 1; T2, treatment 2; T3, treatment 3. *: P < 0.05 versus C (Duncan’s multiple range test).
(TIF)

S3 Fig Reduced bone mineralization in the T3 group. Ash, calcium, and phosphorus content in tibiae were examined after twelve months of bone loss induction. C, control; T1, treatment 1; T2, treatment 2; T3, treatment 3. *: P < 0.05 versus C (Duncan’s multiple range test).
(TIF)
