# Supplementary material for: Porcine induced pluripotent stem cell-derived osteoblast-like cells prevent glucocorticoid-induced bone loss in Lanyu pigs
Source: PLoS One. 2018 Aug 29;13(8):e0202155. doi: 10.1371/journal.pone.0202155 (PMC6114725; doi:10.1371/journal.pone.0202155)
Supplement: S1 Table — (DOCX) [file pone.0202155.s001.docx]

**Supporting information**

**S1 Table. Ingredients of experimental diets.**

| Ingredients | 0.5% calcium diet | 1% calcium diet |
| --- | --- | --- |
| Corn | 759 | 743 |
| Soybean meal | 225 | 225 |
| CaHPO_4_ | 0 | 10 |
| CaCO_3_ | 9 | 15 |
| Salt | 5 | 5 |
| Vitamin premix | 1 | 1 |
| Mineral premix | 1 | 1 |
| Total (kg) | 1,000 | 1,000 |
