# Supplementary figures and images for: Porcine induced pluripotent stem cell-derived osteoblast-like cells prevent glucocorticoid-induced bone loss in Lanyu pigs
Source: PLoS One. 2018 Aug 29;13(8):e0202155. doi: 10.1371/journal.pone.0202155 (PMC6114725; doi:10.1371/journal.pone.0202155)

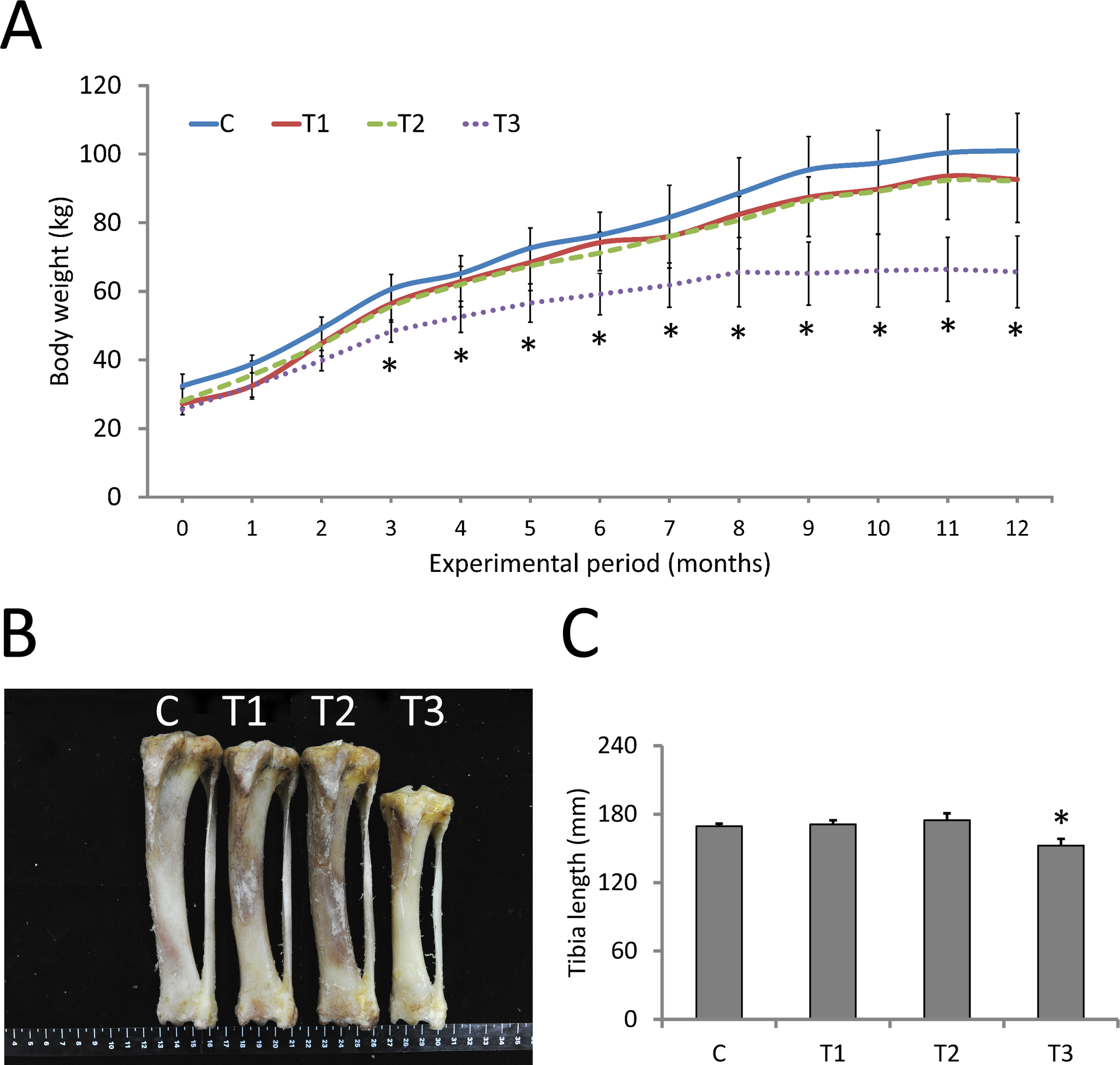

Supplement: S1 Fig — (A) Weight of Lanyu pigs during the experimental period. (B) Representative image of tibiae after twelve months of bone loss induction. (C) Results of tibia length after twelve months of bone loss induction. C, control; T1, treatment 1; T2, treatment 2; T3, treatment 3. *: P < 0.05 versus C (Duncan’s multiple range test). (TIF) [file pone.0202155.s002.tif]

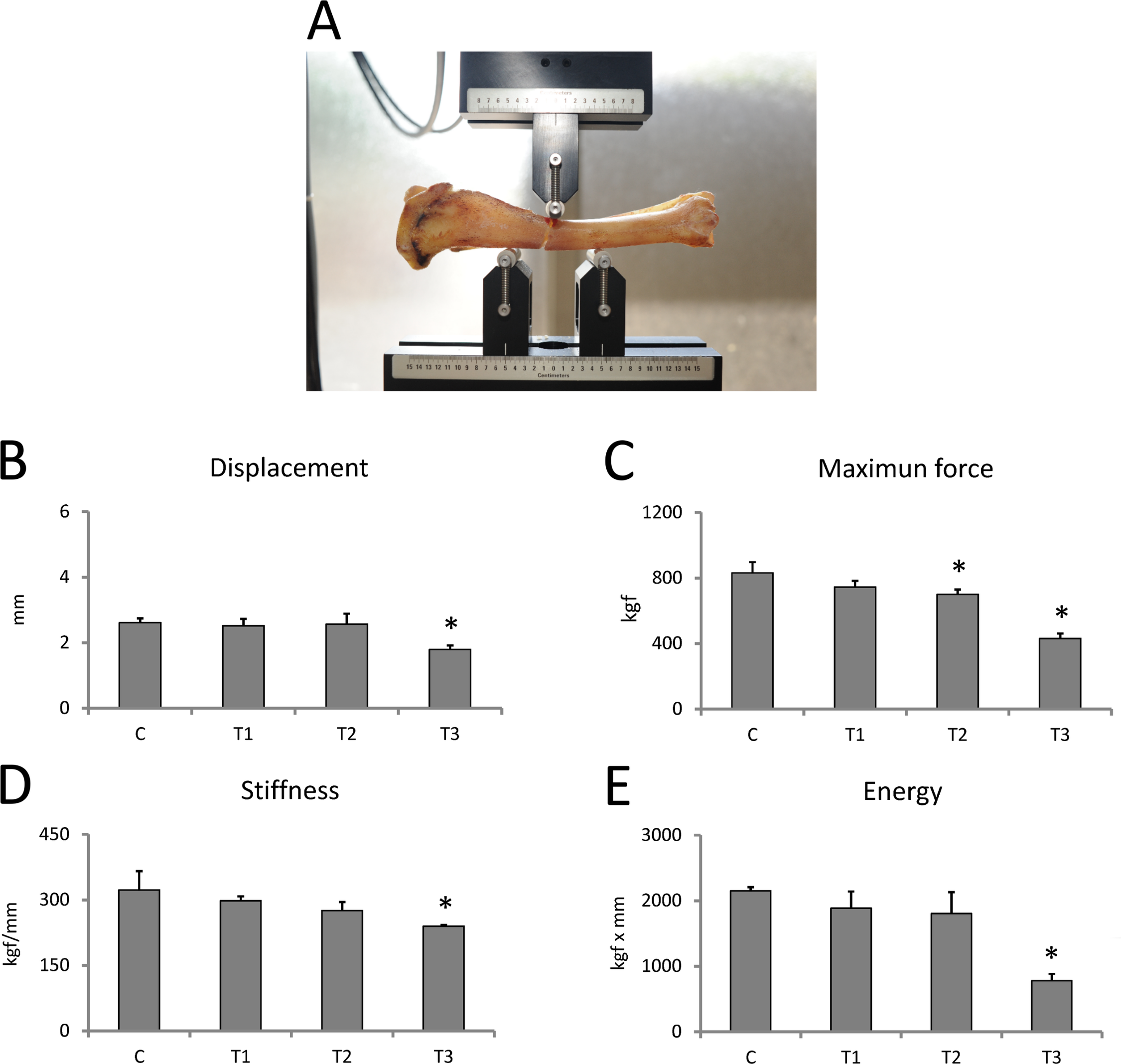

Supplement: S2 Fig — (A) Representative image of tibia on the mechanical test equipment while three-point bending test. (B-E) Quantification of displacement, maximum force, stiffness, and energy parameters. C, control; T1, treatment 1; T2, treatment 2; T3, treatment 3. *: P < 0.05 versus C (Duncan’s multiple range test). (TIF) [file pone.0202155.s003.tif]

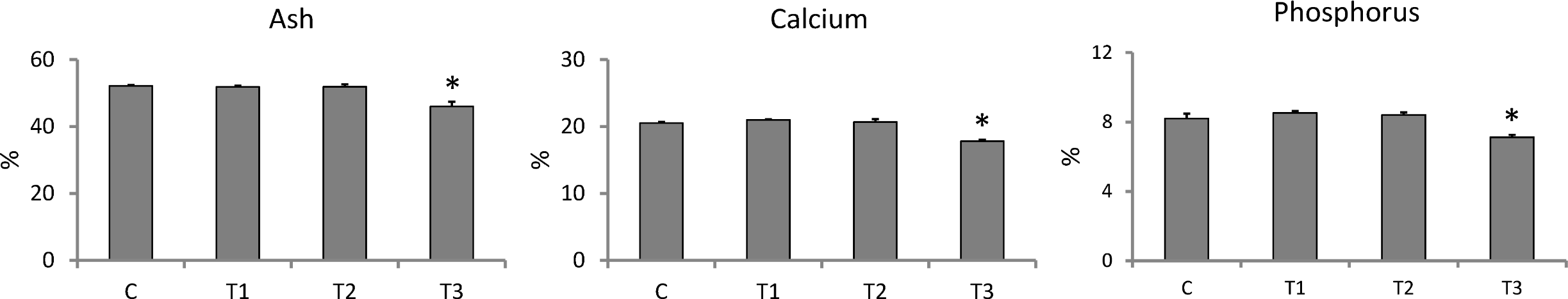

Supplement: S3 Fig — Ash, calcium, and phosphorus content in tibiae were examined after twelve months of bone loss induction. C, control; T1, treatment 1; T2, treatment 2; T3, treatment 3. *: P < 0.05 versus C (Duncan’s multiple range test). (TIF) [file pone.0202155.s004.tif]
